# Urine Analysis as a Reliable Indicator of a Urinary Tract Infection: A Cross-Sectional Study

**DOI:** 10.7759/cureus.101074

**Published:** 2026-01-08

**Authors:** Zaraq R Khan, Zeeshan Ullah, Adel Alwakeedi, Imad Majeed, James Snyder

**Affiliations:** 1 Infectious Disease, University of Louisville Hospital, Louisville, USA; 2 Internal Medicine, Lady Reading Hospital, Peshawar, PAK; 3 Microbiology, University of Louisville Hospital, Louisville, USA

**Keywords:** antibiotic stewerdship, asymptomatic bacteriuria (asbu), sterile pyuria, urinary tract infection, uti criteria

## Abstract

Background: Urinary tract infection (UTI) is one of the most common clinical entities encountered by physicians in a healthcare setup. The fact that there are no definitive criteria for diagnosing a UTI, leads to overdiagnosis and hence excessive use of antibiotics which eventually leads to multi-drug resistant microorganisms.

Objectives: To determine the correlation of positive urine analysis based on greater than 10 white blood cell count (WBC)/high power field (HPF) with a true UTI according to our operational definition.

Methods: This study is a cross-sectional retrospective study. The patient population to be studied consists of in-patients and patients being evaluated in the emergency room in the University of Louisville Healthcare (UofL Health) network. A target of 100 patients was set as a goal for our study. A non-randomized consecutive sampling was done. Data were analyzed using the Statistical Package for the Social Sciences (SPSS) version 25. Descriptive statistics, including means ± SDs for quantitative variables and frequencies/percentages for qualitative variables, were computed.

Results: Our study included 100 patients, with a gender distribution of 48% male and 52% female, and a mean age of 59.62 years (SD = 17.56, range: 20-94 years). UTI diagnoses were established in 17% of cases as per our operational definition in which the majority were female (76%). Symptomatic presentation was observed in 23% of the cohort, while the majority (77%) remained asymptomatic.

Conclusion: Urine analysis is not always a true indicator of a UTI and hence hospital policy of doing a reflex culture to a positive urine analysis should be discouraged as it leads to overdiagnosis and, hence, unnecessary use of antibiotics.

## Introduction

Urinary tract infection (UTI) is one of the most commonly treated infections in clinical practice, accounting for approximately 0.9% of all ambulatory visits in the United States [1]. Each year approximately 150 million people develop a UTI [2]. The clinical phenotypes of UTIs vary from uncomplicated cystitis to complicated infections such as pyelonephritis and perinephric abscesses [3]. The fact that it is a common entity makes it prone to overdiagnosis in healthcare settings across the globe, which eventually leads to more antibiotic usage and consequently more antimicrobial drug resistance [4]. The definition of a UTI varies considerably [5]. One of the essential components of the criteria is the number of leukocytes seen in a urine sample. Greater than or equal to 10 pus cells per high power field (HPF) is considered the minimum which should alert the physician of a possible UTI [5]. This limit increases the sensitivity of testing but significantly decreases the specificity [6]. Some hospitals have implemented a policy of doing a reflex urine culture whenever there are more than 10 leukocytes in a urine analysis without regard to patient symptoms. This not only results in the overuse of resources but also increases the inadvertent use of antibiotics. Our study aims to determine the correlation of a positive urine analysis to UTI in patients presenting to the University of Louisville Healthcare (UofL Health) setup.

## Materials and methods

Study design and setting

This cross-sectional retrospective study was conducted within the UofL Health network. The study population consisted of in-patients and patients evaluated in the emergency room. All laboratory testing was centralized at the University of Louisville Hospital, serving as the core laboratory for the healthcare system. Outpatient clinic patients were excluded from this study.

Sample and sampling method

The study targeted a sample size of 100 patients, using a non-probability consecutive sampling method.

Study objectives

The primary objective of the study was to determine the percentage of positive urine analyses, based on more than 10 white blood cell count (WBC)/HPF, that correlated with a true UTI. Secondary objectives included determining the percentage of patients with positive urine analyses who were symptomatic and the percentage of patients with positive urine analyses who also had positive urine cultures.

Exclusion criteria

The exclusion criteria were designed to ensure that the patient population had the capacity to express their symptoms. Patients were excluded if they were unconscious, disoriented, confused, or had severe dementia or intellectual disabilities. Additionally, those with neurological deficits leading to impaired sensation in the lower extremities and/or perineal region were excluded. Patients presenting with symptoms of urethritis, such as visible genital discharge, were also excluded based on the assumption that common organisms involved in these cases are difficult to grow on culture. Furthermore, any patient whose UTI-related symptoms were not assessed during hospital admission, as per their charts, was excluded from the study.

Data collection procedure

Data were retrospectively collected by obtaining a list of all urine analyses with reflex cultures conducted in October 2023 from the microbiology lab. A total of 100 patients were identified, and chart reviews were performed to assess relevant clinical symptoms. For the purposes of this study, a UTI was defined as a clinical syndrome characterized by pyuria and a documented microbial pathogen on urine culture, accompanied by at least two focal representative symptoms, including dysuria, increased frequency, urgency, lower abdominal or flank pain, or systemic symptoms such as a fever exceeding 38°C. It is important to note that fever in isolation, without focal symptoms, was not considered a reliable clinical indicator of a UTI. Urine culture growth of ≥10^4^ colony-forming units per milliliter (CFU/ml) in clean mid-catch samples, and ≥10^3^ CFU/ml in samples taken via a urinary catheter, was considered significant. Growth of three or more organisms in culture was regarded as indicative of contamination. Pyuria was defined as the presence of pus cells greater than 10/HPF.

Data analysis procedure

Data were analyzed using the Statistical Package for the Social Sciences (SPSS) version 25. Descriptive statistics, including means and standard deviations for quantitative variables and frequencies/percentages for qualitative variables, were computed. The presence or absence of UTIs was stratified based on age, gender, pus cell counts, urine culture positivity, and symptomatic presentation. The association between these categorical variables was assessed using chi-square tests, with a significance level set at p ≤ 0.05. Results were communicated through tables and charts to facilitate clear presentation and interpretation.

Ethical considerations

Throughout the study, patient confidentiality was strictly maintained, and no personal information was shared or published. No incentives were provided to the participants. The study received approval from the University of Louisville Institutional Review Board (IRB) and UofL Hospital.

## Results

Association between UTI and pus cell count

The results showed that among cases with pus cell counts in the range of 10-25 cells/HPF, five exhibited UTIs, while 29 did not. In the 26-50 cells/HPF range, one case had a UTI, and 14 did not. Notably, in cases with pus cell counts exceeding 50 cells/HPF, 11 had UTIs, and 40 did not (Figure 2). Chi-square tests were performed to assess the significance of these associations (p-value = 0.36). Table 1 reveals varying distributions of pus cells in urine based on the presence or absence of UTI. Among cases with UTI, the majority exhibited >50 pus cells/HPF (11 out of 17). In contrast, cases without UTI predominantly showed lower counts of pus cells, with 29 out of 83 having 10-25 pus cells/HPF.

**Table 1:** Chi-square test assessing the association between various variables HPF: High power field

Variable	UTI Present (n = 17)	UTI Absent (n = 83)	Total (N = 100)	Test Statistic	p-value
Pus Cell Count (cells/HPF)				χ² = 2.04	0.36
10–25	5	29	34		
26–50	1	14	15		
>50	11	40	51		
Urine Culture				χ² = 7.64	0.006
Culture Positive	17	56	73		
Culture Negative	0	27	27		
Symptomatic Status				χ² = 67.8	< 0.001
Symptomatic	17	6	23		
Asymptomatic	0	77	77		

Our study included 100 patients, with a gender distribution of 48 (48%) males and 52 (52%) females, and a mean age of 59.62 years (SD = 17.56, range: 20-94 years). UTI diagnoses were established in 17 (17%) cases as per our operational definition, with the majority being female (76%). Symptomatic presentation was observed in 23 (23%) of the cohort, while the majority (77 (77%)) remained asymptomatic. Notably, 73 (73%) participants exhibited positive urine cultures. WBC in urine analyses revealed that 51 (51%) exhibited counts exceeding 50 cells/HPF, 34 (34%) fell within the range of 10-25 cells/HPF, and 15 (15%) displayed counts ranging from 26 to 50 cells/HPF (Table 2).

**Table 2:** Basic characteristics and organisms distribution HPF: High power field; UTI: Urinary tract infection; WBC: White blood cell count

Variable	n (%) or Mean ± SD
Total Patients	100
Gender Distribution	
Male	48 (48%)
Female	52 (52%)
Mean Age (years)	59.62 ± 17.56
Age Range (years)	20–94
UTI Diagnosed	17 (17%)
Female (among UTI cases)	13 (76%)
Symptomatic Presentation	
Symptomatic	23 (23%)
Asymptomatic	77 (77%)
Positive Urine Culture	73 (73%)
WBC in Urine (cells/HPF)	
10-25 cells/HPF	34 (34%)
26-50 cells/HPF	15 (15%)
>50 cells/HPF	51 (51%)
Organisms Isolated	
*E. coli*	27 (27%)
*Klebsiella pneumoniae*	10 (10%)
*Enterococcus faecalis*	5 (5%)
*Proteus mirabilis*	3 (3%)
*Staphylococcus*	6 (6%)
Yeast	2 (2%)

**Figure 1:**
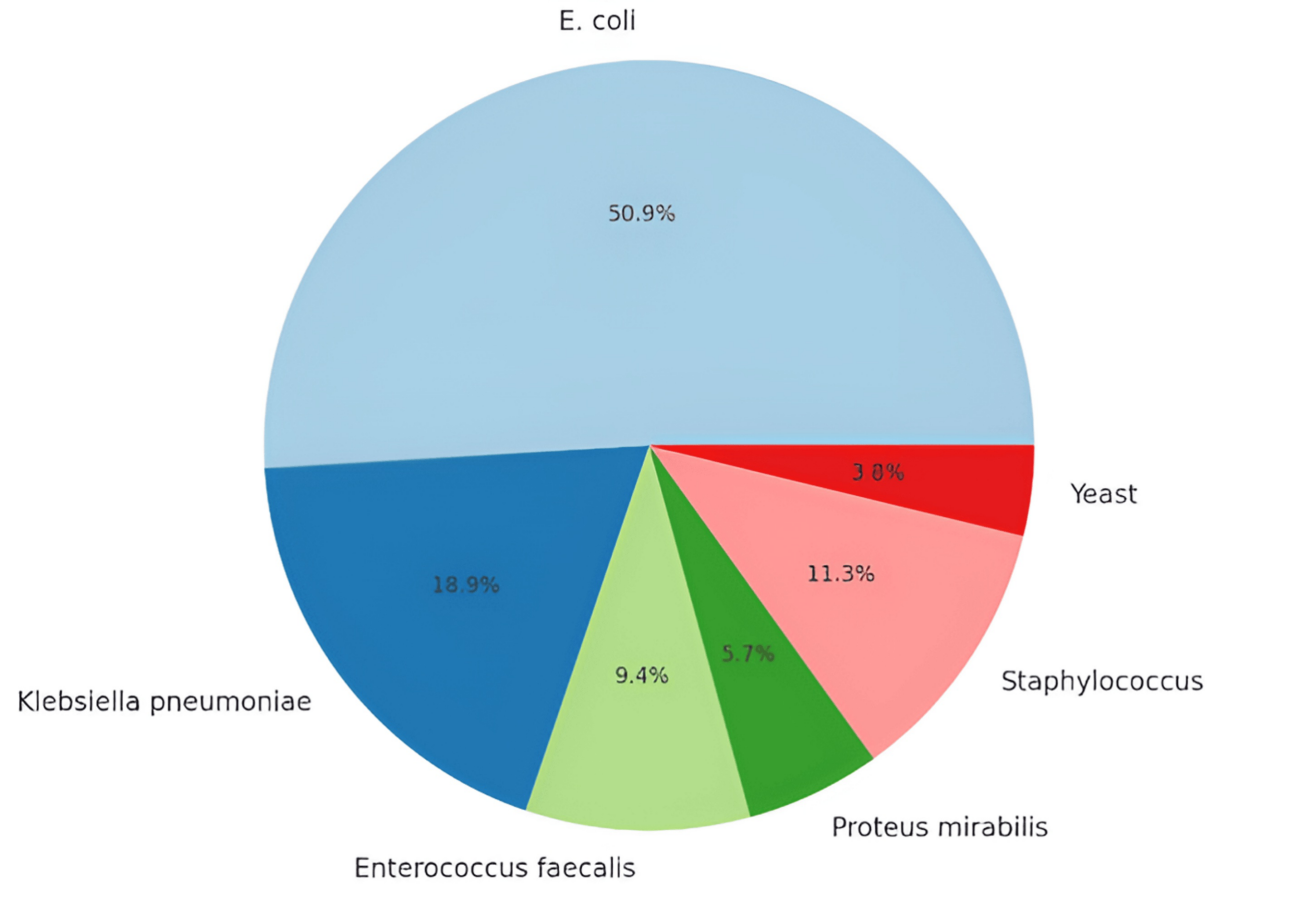
Distribution of organisms in positive urine culture

The organisms isolated from these cultures varied, with *E. coli* being the most prevalent (50.9%), followed by *Klebsiella pneumoniae* (17.9%), *Enterococcus faecalis* (9.4%), *Proteus mirabilis* (5.7%), *Staphylococcus* (11.3%), and Yeast (3.8%) (Figure 1).

Association between positive urine culture and UTI

**Figure 2:**
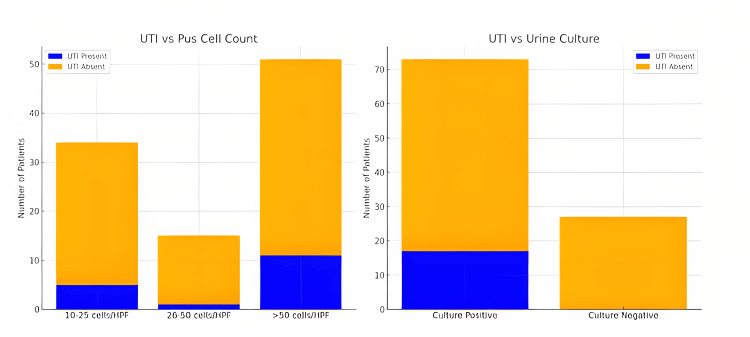
Pus cell count and urine culture status with UTI UTI: Urinary tract infection

An analysis of the relationship between positive urine cultures and the presence of UTIs was conducted through a cross-tabulation analysis. The results showed that among cases where UTI was present, 17 out of 17 had a positive urine culture. Conversely, in cases where UTI was absent, a considerable proportion still exhibited positive urine cultures (56 out of 83) (Figure 2). This unexpected finding suggests that a proportion of individuals without clinically diagnosed UTIs may still present with positive results in urine cultures. The Chi-square test was applied to assess the statistical significance of this association. The results revealed a Pearson Chi-square value of 7.57 with 1 degree of freedom (df), yielding an asymptotic significance (two-sided) of p-value = 0.006 (Table 1).

Association between symptomatic presentation and UTI

An analysis of the association between symptomatic presentation and the presence of UTIs was conducted. The results revealed a clear pattern: among cases with UTIs, all 17 were symptomatic, while none were asymptomatic. Conversely, among cases without UTIs, 77 out of 83 were asymptomatic, and six displayed symptoms. Chi-square tests were employed to evaluate the statistical significance of this association. The Pearson Chi-square value was 67.8 with 1 degree of freedom (df), resulting in an asymptotic significance (two-sided) of p-value < 0.000 (Table 1).

## Discussion

UTIs affect approximately 400 million individuals globally each year [3]. These infections, involving the urethra, bladder, ureters, and/or kidneys, commonly present with symptoms such as dysuria, increased urinary frequency and urgency, fever, and pelvic discomfort [7]. UTIs are associated with significant short-term morbidity, typically lasting six days and impairing work, academic productivity, and personal relationships for at least two of those days [8]. The societal burden is substantial, with UTIs accounting for 13.7% of community antibiotic prescriptions and up to 3% of primary care consultations [9,10]. Epidemiologically, women, particularly those who are young or elderly, are more frequently affected, with one in three women aged 24 having received treatment for a UTI [11]. Hospital-acquired UTIs account for 35-40% of nosocomial infections [12].

In our study, UTIs were operationally defined by the presence of pyuria, positive urine culture identifying a microbial pathogen, and at least two characteristic symptoms. Based on these criteria, the prevalence of UTI in our study population was 17%.

The diagnostic role of pyuria is well acknowledged, and our findings support its continued use, although the association with culture-confirmed UTIs was not statistically significant (p = 0.36). This underscores the diagnostic complexity of UTI and necessitates a critical appraisal of the clinical value of pus cell quantification. Literature suggests that while pyuria (defined as >10 WBC/HPF or a positive leukocyte esterase) can indicate inflammation, it lacks specificity for infection [13,14]. Furthermore, centrifuged urine samples with ≥5 WBC/HPF are generally considered clinically significant, yet thresholds vary across studies [15-18].

In our investigation, we categorized pyuria into three groups: 10-25, 26-50, and >50 pus cells per high-power field. Notably, most individuals with counts >50 met our clinical UTI criteria. However, this stratification did not yield statistical significance, prompting consideration of pyuria’s limited predictive value outside of a comprehensive clinical and microbiological context.

All 17 individuals diagnosed with UTI had positive urine cultures and exhibited symptoms, reaffirming the need to pair microbiological findings with clinical presentation. Urine cultures, though regarded as diagnostic gold standards, carry risks of contamination and false positives [17,18]. The high rate of positive cultures among patients not meeting UTI criteria (56 out of 83) suggests potential overdiagnosis or colonization rather than active infection. The significant statistical correlation (p = 0.006) prompts critical evaluation of reliance on culture positivity in isolation.

Indeed, literature confirms that bacteriuria does not invariably equate to symptomatic infection, and conversely, some symptomatic individuals may lack culture-confirmed bacteriuria [19,20]. This dichotomy complicates diagnosis and calls for a nuanced, patient-centered approach, particularly in recurrent UTI cases [21,22].

The strong association between symptomatic presentation and UTI (p < 0.001) supports our operational definition. However, asymptomatic bacteriuria-especially prevalent in the elderly-poses a clinical dilemma. Six of 83 asymptomatic individuals had both positive cultures and elevated pus cells. The Infectious Diseases Society of America (IDSA) discourages routine screening and treatment of asymptomatic bacteriuria except in pregnant women or patients undergoing urological procedures [23,24].

Conversely, many individuals without UTI also exhibited pyuria and symptoms, creating a diagnostic gray zone. Literature suggests that combinations of findings-such as WBC >10/HPF, positive nitrites, and bacterial index ≥4-enhance diagnostic accuracy [24].

Our findings, grounded in a robust operational definition, emphasize the complexity of UTI diagnosis. The presence of asymptomatic bacteriuria and the limited utility of pus cell counts challenge traditional diagnostic paradigms. Further research should explore microbial colonization patterns and refine diagnostic thresholds to balance sensitivity with specificity.

Limitations

This study’s retrospective design and narrowly defined operational criteria may limit generalizability. The exclusion of variables such as comorbid conditions is a limitation that must be considered when extrapolating findings to broader populations.

## Conclusions

Our study found that a significant proportion of patients with positive urine analysis results did not meet the criteria for a true UTI, with only few of positive urine analyses correlating with clinical UTIs. This suggests that the current hospital policy of performing reflex cultures on all patients with positive urine analyses may lead to overdiagnosis and unnecessary antibiotic use. These findings underscore the need for a more targeted approach in diagnosing UTIs, which could enhance cost-effectiveness and reduce the risk of antibiotic resistance. Implementing stricter criteria for reflex culture testing could better align laboratory practices with clinical needs, improving patient care and resource utilization.
